# Editorial: On Tissue Engineering and Regenerative Medicine of Skin and Its Appendages

**DOI:** 10.3389/fphys.2019.00712

**Published:** 2019-06-11

**Authors:** Marianna Bei, Basak E. Uygun

**Affiliations:** ^1^Department of Surgery, Harvard Medical School, Boston, MA, United States; ^2^Center for Engineering in Medicine, Massachusetts General Hospital, Boston, MA, United States; ^3^Research Department, Shriners Hospitals for Children, Boston, MA, United States

**Keywords:** skin, stem cells, wound healing, skin substitutes, inflammation, biomateials

This Frontiers research topic, on tissue engineering and regenerative medicine of skin and its appendages, covers mainly cellular, molecular, and engineering aspects of the pathology, repair, and/or regeneration of skin after wound induction. Chronic or non-healing wounds represent a growing health problem worldwide. The underlying pathogenesis of chronic wounds is further complicated by factors such as advanced age, poor nutrition, and immunosuppression which put additional cellular and systemic stress and thus, delaying wound healing. The current treatments do not allow the skin tissue to fully regenerate and the recurrence rate of chronic wounds is extremely high. Thus, there is a significant need for improved understanding of the biology occurring during wound healing process and identify targeted therapies that can yield satisfactory clinical outcomes.

The topic presents 7 manuscripts and 2 reviews from different countries that together convey important aspects of skin wound healing research. As it is impossible, in this short editorial, to highlight all aspects of each of the papers and reviews submitted to this topic, we have chosen to present an overview of the main findings for each paper and an overall perspective of the two state of the art reviews included in this digital collection. Specifically:

The study on the reparative abilities of menstrual stem cells (MenSCs) to modulate the wound matrix signals and improve cutaneous regeneration by Cuenca et al. compares the biological functions and the transcriptomic pattern of MenSCs with umbilical cord MSCs. Their findings provide evidence, for the first time, about the superior clonogenicity, immunosuppressive, migration, and enhanced wound healing potential of MenSCs compared to that of MSCs. A valuable source of MSCs is the gelatinous material of the umbilical cord called Wharton's jelly. WJ-derived MSCs have been the subject of extensive research, while very little is known about the de-cellularized jelly material of the umbilical cord, a native niche of stem cells. Bakhtyar et al. explore the role of acellular Wharton's jelly. They isolate Wharton's jelly from umbilical cords and then fractionate acellular gelatinous Wharton's jelly (AGWJ) providing evidence on the role of AGWJ in enhancing wound healing *in vitro* and *in vivo*. The research paper by Wang et al. on the other hand, highlights the role of a specific neuronal protein, that of P311, in promoting cutaneous wound healing and regeneration. P311 is expressed by the endothelial cells in the dermis of murine and human skin wounds and, when its function is eliminated either *in vitro* or *in vivo*, wound healing is delayed due to reduction of angiogenic factors such as VEGF and TGFβ1. These findings suggest that P311 may play a significant role in angiogenesis during wound healing.

The study by Kravez et al. opens new possibilities for quantitative and more objective assessment of scar severity. So far, scar size prediction is based on imaging modalities that lack the ability to image individual layers of the scar. The authors develop a predictive model of scar formation based on polarization sensitive optical frequency domain imaging (PS-OFDI), which offers comprehensive subsurface imaging and predicts the size of a scar 6 months after third-degree burn injuries in rats. The importance of accurately predicting scar size comes as an imperative necessity in case of keloid formation after wound induction, a fibrotic skin condition that typically results from abnormal wound healing, and is characterized by the deposition of an excess of extracellular matrix (ECM). The study by Jiao et al. provides a thorough investigation of the histology and biology of keloid lesion at different depths, focusing on the cellular composition, collagen composition and the biological behavior of the fibroblasts at the different levels within the keloid dermis. A key finding of this study is the identification of the superficial dermis layer as the initiator of keloid formation, providing thus a cue in which layer intra-lesional injection of pharmaceuticals and of other treatments should be performed for keloid.

The role of engineering approaches to improve skin wound healing using synthetic, biopolymeric scaffolds loaded with different therapeutic and antimicrobial agents, is very popular. In this topic, Wang et al. introduce the formation of composite nanofibrous membranes using chitosan (CS) and poly (vinyl alcohol) (PVA) loaded with antibiotics at different ratios fabricated by electrospinning. The authors show that there is a specific volumetric ratio of CS/PVA to achieve an optimal nanofibrous structure and that the CS/PVA electrospun scaffold has great potential to be used for infection related wound dressing and thus for skin tissue regeneration. On the other hand, the paper by Chung et al. introduces selenium nanoparticles as novel antibiotic chemical composites to which there is no known bacterial resistance. Using this unique property, they dope selenium nanoparticles to electrospun silk scaffolds to impart antibacterial properties to silk and show for the first time that these scaffolds reduce bacterial growth *in vitro*.

The research findings presented in this topic have as major goal to stimulate our thinking and open new avenues of research. Both, clinicians and researchers will find up-to-date discoveries and perspectives on the etiology, translational, and therapeutic approaches for skin wound healing. The current status of research however is elegantly summarized in the two state of the art reviews that report ongoing efforts to improve the outcomes of chronic wound healing therapies. The review by Krzyszczyk et al. explains different macrophage phenotypes *in vivo* and *in vitro* as described in the literature and discusses the attempts to restore the macrophage function by therapeutic approaches. It summarizes experimental therapies that aim to attenuate endogenous M1 macrophages, supplement exogenous M2 macrophages, or use cells and growth factors that modulate the macrophage transition from M1 to M2 phenotype. The authors emphasize the need to standardize the macrophage phenotype nomenclature and definition to provide consistency in the field and improve the understanding of their roles to promote chronic wound healing. The second review is by Dickinson and Gerecht who provide a comprehensive overview of skin substitutes that are in clinical use. A challenge in using engineered skin substitutes for treatment of chronic wounds is that there is no consensus of one significantly outperforming the others. Recent research efforts aim to develop innovative materials that have the ability modulate the endogenous cellular processes to promote wound healing. The review summarizes these efforts focusing on synthetic and polymeric materials as scaffolds that have the advantage of tunable mechanical and biological characteristics for enhanced wound healing.

Space precludes a more detailed description of the articles and reviews published in this topic but readers are urged to visit the on line content list to quickly access the full papers. We would like to thank the team at Frontiers for allowing open access to the papers as soon as they are accepted for publication following full peer review. We also thank the contributors whose dedication to their science brought this research topic to fruition. We hope that the research presented here continues to develop and advances our understanding of skin regeneration and wound healing research.

## Author Contributions

All authors listed have made a substantial, direct and intellectual contribution to the work, and approved it for publication.

### Conflict of Interest Statement

The authors declare that the research was conducted in the absence of any commercial or financial relationships that could be construed as a potential conflict of interest.

